# Targeting the MDM2-p53 pathway in dedifferentiated liposarcoma

**DOI:** 10.3389/fonc.2022.1006959

**Published:** 2022-11-10

**Authors:** Raymond S. Traweek, Brandon M. Cope, Christina L. Roland, Emily Z. Keung, Elise F. Nassif, Derek J. Erstad

**Affiliations:** ^1^ Department of Surgical Oncology, The University of Texas MD Anderson Cancer Center, Houston, TX, United States; ^2^ Division of Surgical Oncology, Baylor College of Medicine, Houston, TX, United States

**Keywords:** dedifferentiated liposarcoma, MDM2, p53, targeted therapy, clinical trial, small molecular inhibitor

## Abstract

Dedifferentiated liposarcoma (DDLPS) is an aggressive adipogenic cancer with poor prognosis. DDLPS tumors are only modestly sensitive to chemotherapy and radiation, and there is a need for more effective therapies. Genetically, DDLPS is characterized by a low tumor mutational burden and frequent chromosomal structural abnormalities including amplification of the 12q13-15 chromosomal region and the *MDM2* gene, which are defining features of DDLPS. The MDM2 protein is an E3 ubiquitin ligase that targets the tumor suppressor, p53, for proteasomal degradation. *MDM2* amplification or overexpression in human malignancies is associated with cell-cycle progression and worse prognosis. The MDM2–p53 interaction has thus garnered interest as a therapeutic target for DDLPS and other malignancies. MDM2 binds p53 *via* a hydrophobic protein interaction that is easily accessible with synthetic analogues. Multiple agents have been developed, including Nutlins such as RG7112 and small molecular inhibitors including SAR405838 and HDM201. Preclinical *in vitro* and animal models have shown promising results with MDM2 inhibition, resulting in robust p53 reactivation and cancer cell death. However, multiple early-phase clinical trials have failed to show a benefit with MDM2 pathway inhibition for DDLPS. Mechanisms of resistance are being elucidated, and novel inhibitors and combination therapies are currently under investigation. This review provides an overview of these strategies for targeting MDM2 in DDLPS.

## Introduction

Liposarcomas are rare, adipogenic cancers that typically arise in the extremities and retroperitoneum. They are composed of several histologic subtypes, of which well-differentiated and dedifferentiated tumors are the most common. Well-differentiated liposarcomas (WDLPS) exhibit a more indolent behavior with frequent local recurrence. Dedifferentiated liposarcomas (DDLPS) are of a higher grade with potential for distant metastasis (1). DDLPS can arise *de novo*, in mixed states concomitantly with WDLPS, or upon recurrence after resection of WDLPS (2).

Surgical resection remains the definitive management for DDLPS in appropriate candidates. However, due to the frequent invasion of surrounding structures and the large size of these tumors, achieving a microscopically negative resection margin remains difficult. Local recurrence is common, and perioperative therapies are frequently utilized (3, 4). First-line chemotherapy is anthracycline based, usually doxorubicin in combination with ifosfamide. Unfortunately, DDLPS are relatively chemoresistant, with radiographic responses observed in less than one-third of patients (5). Consequently, there is no consensus regarding systemic therapy in the neoadjuvant or adjuvant setting. Preoperative radiation therapy is frequently utilized in academic treatment centers for lower-grade tumors, as it may reduce local recurrence (6, 7). Overall, outcomes remain poor with high rates of recurrence and a limited overall survival of approximately 50% following the first local recurrence (8–10). For these reasons, there has been increasing interest in targeted therapies in the treatment of DDLPS.

The tumor biology of DDLPS is characterized by a low tumor mutational burden (TMB) with high somatic copy-number alterations (SCNAs) (11). Amplification of chromosome 12q13-15 results in amplification of *MDM2*, a defining feature of DDLPS (12). The MDM2 protein has E3 ubiquitin ligase activity that targets the tumor suppressor p53, for degradation. Overexpression of the MDM2 protein is associated with cell-cycle progression and malignant proliferation, thus offering a potential treatment target. While MDM2 inhibitors have been associated with promising preclinical findings, results from early-phase clinical trials have thus far been disappointing (13, 14). Nonetheless, MDM2 inhibition remains an area of ongoing investigation, including the development of novel inhibitors and combination therapies (15, 16).

In this review, we discuss the unique genetic and epigenetic characteristics of DDLPS and the rationale for targeting MDM2. We build on this information by describing the MDM2–p53 pathway and provide a summary of approaches to MDM2 drug design. Finally, we review preclinical and clinical trials focused on MDM2 inhibition, examining mechanisms of failure and future directions for improved pathway inhibition and therapeutic efficacy.

## The genomic landscape of DDLPS

DDLPS are characterized by a mutational signature defined by 12q13-15 amplification, as well as dysregulated epigenetic patterns associated with downregulation of tumor suppressors and increased expression of proliferative genes. While most DDLPS occur *de novo*, they can also present with WDLPS in mixed states for both primary and recurrent tumors. Dedifferentiated tumors are histologically characterized by a transition from mature adipocytes to cells with marked atypia (17). The molecular pathogenesis of dedifferentiation remains poorly understood, although it is of great interest to researchers given its association with adverse tumor biology and worse prognosis.

The genomic landscape of DDLPS is characterized by low TMB and frequent SCNAS. TMB has been identified as a predictive biomarker for response to immunotherapy, supported by the premise that highly mutated tumors are more likely to have detectable neoantigens, although this relationship is not reliable for all cancers (18, 19). In a recent analysis, Chalmers et al. performed whole exome sequencing across multiple cancer types to characterize TMB (20). In this study, undifferentiated soft-tissue sarcomas demonstrated low TMB with a median of 2.5 mutations/Mb, placing them in the bottom 40th percentile among 167 tumor types. These findings were recapitulated by Liu et al., in which a median TMB of 1.97 mutations/Mb was demonstrated for a cohort of DDLPS (21). Accordingly, immune checkpoint blockade with anti-CTLA-4 monotherapy has yielded minimal tumor response in both preclinical and clinical models for DDLPS, which may be partly related to low mutational burden (22, 23).

**Figure 1:**
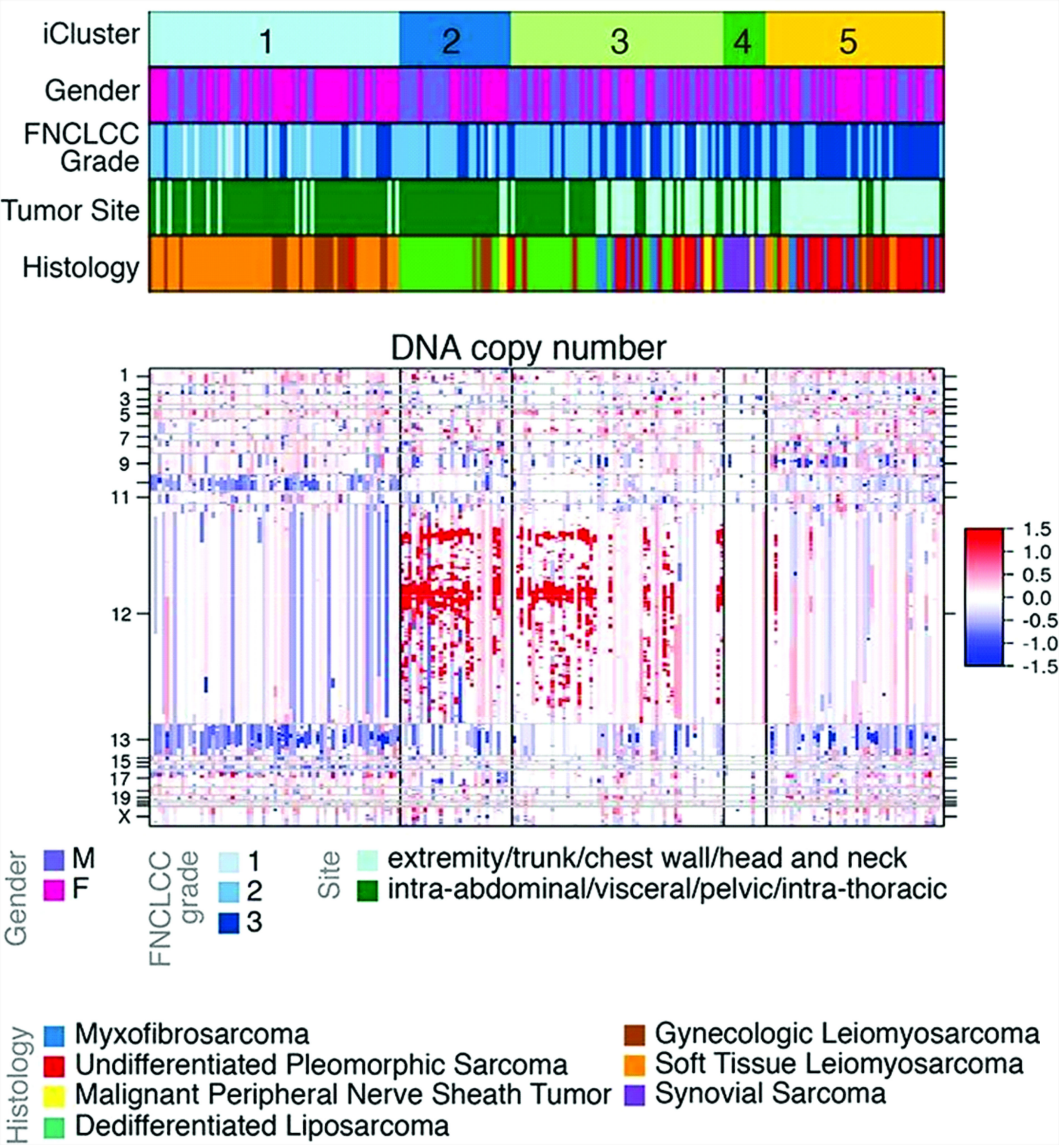
Clustered analysis of DNA copy number among soft-tissue sarcomas. Cluster C1 is primarily composed of leiomyosarcoma. Clusters C2 and C3 are primarily composed of dedifferentiated liposarcoma. Cluster C4 is composed of synovial sarcomas and malignant peripheral nerve sheath tumors. Cluster C5 is composed of high-grade undifferentiated pleomorphic sarcomas. Amplification in red, deletion in blue. Adapted from *Comprehensive and Integrated Genomic Characterization of Adult Soft Tissue Sarcomas* (11).

Compared with other liposarcoma histologic subtypes, DDLPS demonstrates the highest frequency of SCNAs (11). SCNAs have been shown to support tumorigenesis and mutagenic processes that drive genomic instability during tumor growth and evolution (24). Certain patterns of SCNAs appear conserved across different cancers. In a recent pooled analysis of focal SCNAs among 17 tumor types, Beroukhim et al. demonstrated a median overlap of 79% across epithelial, gastrointestinal, and genitourinary malignancies (25). For WDLPS and DDLPS, the highly recurrent focal amplification of chromosome 12q13-15 is a defining SCNA that appears unique to these histologic subtypes (**Figure 1**). Amplification of the 12q arm may be related to incurred chromosomal structural abnormalities, and initial work to characterize the cytogenetics of retroperitoneal liposarcomas by Dal Cin et al. identified supernumerary rings and long marker chromosomes (“rods”) as the primary karyotypic abnormalities within their cohort of WDLPS and DDLPS (11, 26, 27). A subsequent work by Pedeutour et al. demonstrated that these ring and rod structures contained amplified segments of the 12q chromosome (28). Taken together, the finding of abnormal ring/rod chromosomal structures containing amplification of the 12q13-15 arm is a defining feature for WDLPS and DDLPS.

**Figure 2:**
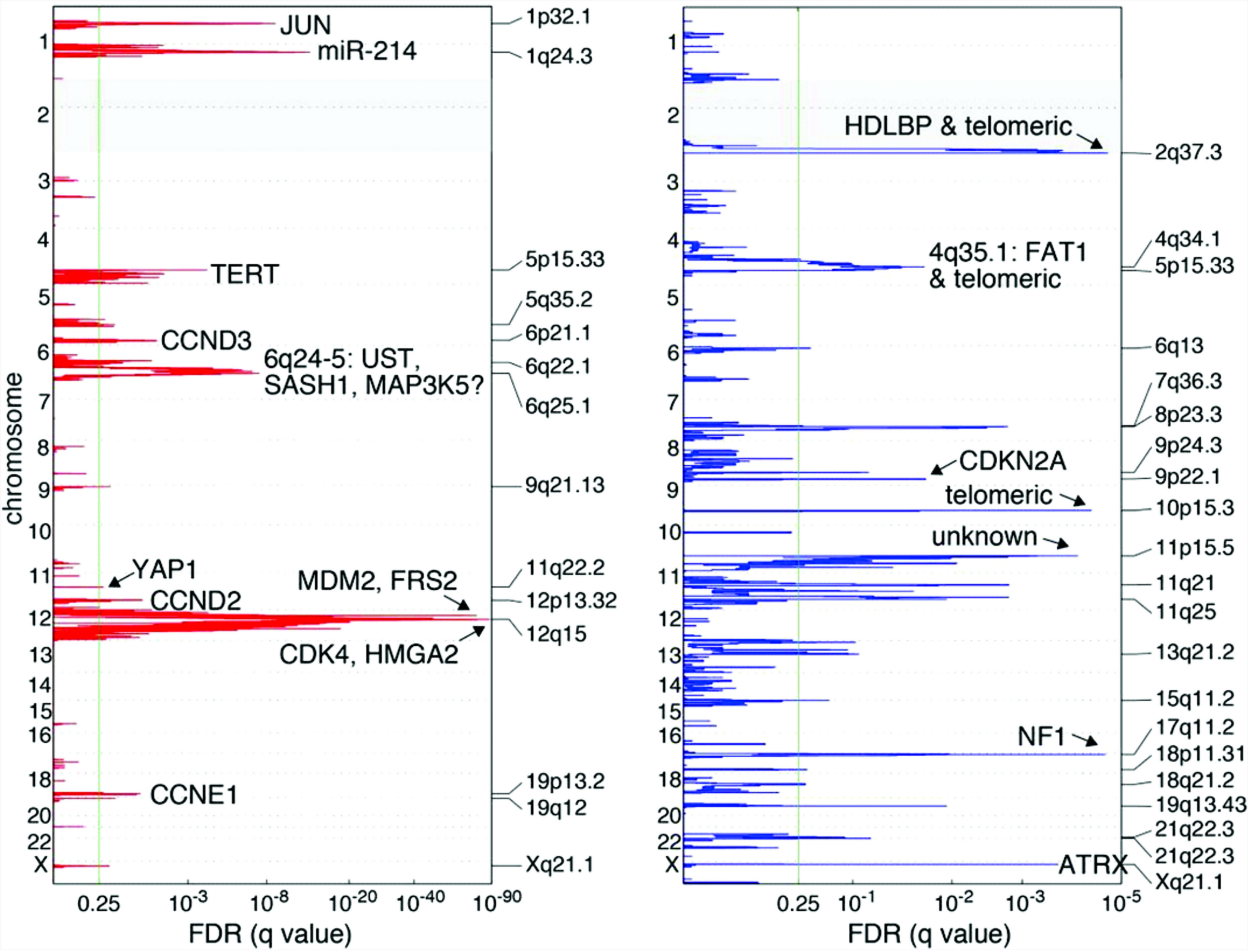
Recurrent genetic alterations among 50 DDLPS samples. Amplification in red, deletions in blue. Green line indicates the significance threshold for focally amplified or deleted genes. Adapted from *Comprehensive and Integrated Genomic Characterization of Adult Soft Tissue Sarcomas* (11).

Subsequent investigations have focused on individual genes located within the 12q13-15 arm, including *MDM2*, *CDK4*, *HMGA2*, and *YEATS4* (**Figure 2**) (29). Of these, *MDM2* is a known oncogene amplified across multiple cancer types (30, 31). Oliner et al. identified a 5–50-fold *MDM2* amplification in both WD and DD LPS with 12q amplification, which has been recapitulated in other studies (12, 31, 32). *MDM2* encodes an E3 ubiquitin ligase that negatively regulates the tumor suppressor p53 by marking it for proteasomal degradation, inhibiting cell-cycle arrest and apoptosis in response to DNA damage (33). Loss of p53 function is a crucial step in malignant transformation for multiple cancer types and has been shown to adversely impact tumor biology (34, 35). Therefore, the MDM2–p53 interaction is a target of interest in cancer therapeutics.

The quantity of 12q13-15 amplification and *MDM2* upregulation appears to be associated with the degree of tumor dedifferentiation (36). Horvai et al. analyzed 29 WD and DD liposarcomas, the majority of which were retroperitoneal with concomitant WD and DD components (37). They observed amplification of 12q13-15 in all tumors, with dedifferentiated components having significantly more total amplifications by comparative genomic hybridization. Similarly, in studies using FISH analysis, *MDM2* amplification was identified in abnormal ring/rod structures, and the quantity of protein expression was associated with the degree of cellular atypia (27). The association between 12q13-15/*MDM2* amplification and dedifferentiation raises the question of whether this mutation supports the dedifferentiation process. Beird et al. analyzed 17 liposarcomas with both WD and DD components and observed an overlap in 8.3% of somatic mutations, suggesting shared clonal ancestry (38). Furthermore, it has been shown that WDLPS frequently precedes dedifferentiation, which may be in part due to the subsequent amplification of *c-Jun*, a proto-oncogene involved in adipocyte differentiation that is primarily observed in ring and rod structures associated with DDLPS (39, 40). Thus, it is reasonable to hypothesize that the phenotypic sequelae from 12q13-15 amplification impact dedifferentiation programs.

Given the low TMB observed in DDLPS, it is thought that epigenetic changes such as aberrant methylation might have an important role in disease pathogenesis. Demicco et al. performed an unsupervised clustering analysis of 50 DDLPS tumor samples and identified hyper- and hypomethylated phenotypes (11). Disease-specific survival (DSS) was significantly worse in association with hypermethylated tumors, suggesting a potential role as an adverse prognostic biomarker. Hypermethylation of genes and proteins affecting adipocyte differentiation has been identified within DDLPS. Taylor et al. demonstrated methylation of *CEBPA*, a core promoter of adipocyte differentiation, within 24% of 80 dedifferentiated tumors, and this was not observed in the analyzed WDLPS tumors or non-neoplastic adipocyte tissue controls (41). The authors confirmed that *CEBPA* hypermethylation was associated with a statistically significant reduction in identifiable *CEBPA* mRNA. Additional work by Keung et al. identified trimethylation of histone H3 at the lysine 9 residue (H3K9me3) among DDLPS cells, which was not observed in WDLPS (42). The authors demonstrated an association between H3K9me3 and downregulation of *KLF6*, a known tumor suppressor, suggesting that histone modification may play a role in driving oncogenesis.

Based on the observation that dedifferentiation is associated with both higher rates of DNA methylation and greater quantity of *MDM2* amplification, it is conceivable that *MDM2* might influence epigenetic modification processes. In this regard, Cao et al., using non-sarcomatous cell lines *in vitro*, observed an inverse association between MDM2 and HBP1 protein expression by IHC (43). HBP1 is a transcription factor that acts as an epigenetic regulator *via* both activator and repressor roles of several key cell-cycle regulator genes. High-affinity binding elements within HBP1 repress cell-cycle regulator genes *N-MYC*, *C-MYC*, *DNMT1*, *and EZH2* at the transcriptional level, whereas downstream effector targets of HBP1 activate genes encoding p21, p16, and histone H.1 (44–48) In this study, the authors identified MDM2-mediated ubiquitination and proteasomal degradation of HBP1, which was associated with increased expressions of DNMT1 and EZH2, resulting in global DNA and histone hypermethylation with subsequent genomic instability. These findings provide an example of a potential mechanism for MDM2-driven epigenetic modification. However, there remains a paucity of information regarding MDM2 activity on epigenetic modifications in DDLPS. In a separate study, Stocker et al. characterized genomic signatures, gene mutations, and methylation profiles for a small sample of DDLPS patients. They identified *MDM2* amplifications in all cases but observed hypermethylated genomes in only 30% of tumors, and there was no significant association between methylation status and mutational signatures (49). Given these observations, further investigation to better characterize potential interactions between MDM2 hyperfunction and epigenetic modifications in DDLPS is warranted.

In addition, it remains difficult to associate epigenetic chromatin modifications with downstream transcriptional changes *in vivo*, as the two often appear unrelated. Moreover, medications that alter DNA methylation status, such as histone deacetylase inhibitors (HDACs), have provided only modest clinical benefit in soft-tissue sarcomas in early-phase clinical trials despite efficacy in hematologic malignancies (50–52). Ultimately, the epigenomics driving the dedifferentiation of liposarcomas are poorly understood and remain an area of ongoing research (53).

Finally, it is worth noting that DDLPS are genetically distinct from pleomorphic liposarcomas (PLPS), although both exhibit dedifferentiated histology and aggressive behavior. PLPS are mutationally characterized by high aneuploidy and complex karyotypic rearrangements (54–56). Unlike WDLPS/DDLPS, amplification of the 12q13-15 region is not a defining feature, although somatic mutations in *TP53* have been observed in up to 17% of cases (17, 56). Barretina et al. evaluated genomic alterations in 24 PLPS compared with 50 DDLPS tumors (26). In this study, pleomorphic tumors demonstrated significant chromosomal alterations and somatic mutations in *NF1*, *RB1*, and *PIK3CA*, but they lacked 12q13-15 amplification (57). In another study, Schmidt et al. evaluated 36 patients with multiple liposarcoma histologic subtypes including WDLPS, DDLPS, myxoid, and PLPS (58). Using comparative genomic hybridization, the authors demonstrated that PLPS tumors contained frequent genomic imbalances occurring in almost all chromosomal regions. In particular, PLPS has been shown to frequently contain copy number gains of chromosomes 1, 5q, 20q, and 22q (59). Clinically, PLPS are more chemosensitive than DDLPS but appear to have less tumor immune infiltration (60–63). Both tumor types have a similar propensity for local recurrence, but pleomorphic LPS are at a higher risk for distant dissemination (64). Importantly, due to the lack of 12q13-15 amplification, pleomorphic sarcomas are not good candidates for targeted MDM2 inhibition.

## Mechanism of the MDM2–p53 protein interaction

**Figure 3:**
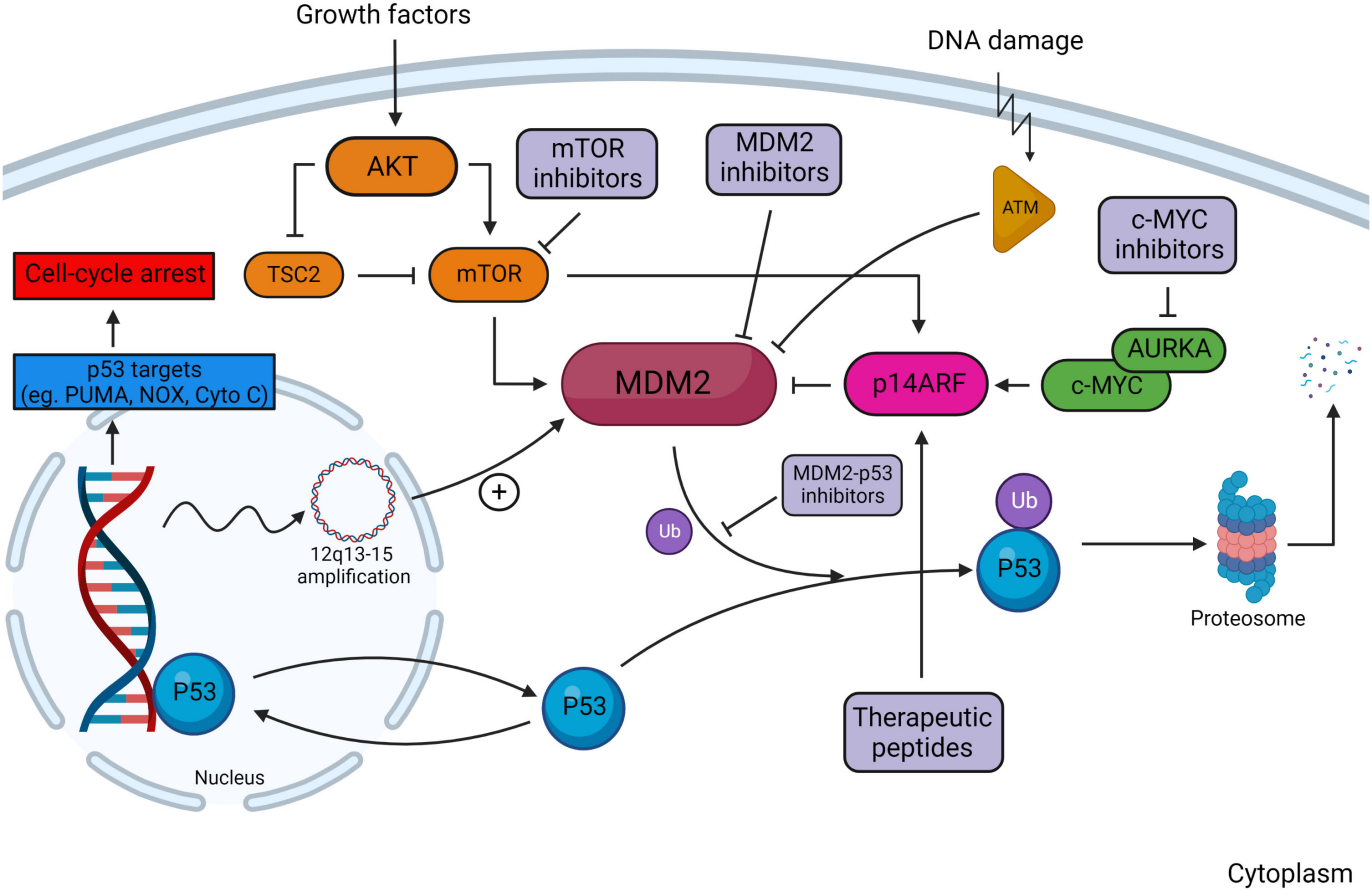
Overview of the MDM2–p53 pathway in response to DNA damage. In the absence of DNA damage, MDM2 facilitates ubiquitination of p53, tagging it for proteosomal degradation. Damage to DNA activates kinases, including ataxia telangiectasia-mutated (ATM) kinase, which are responsible for phosphorylating MDM2 and reducing its affinity to bind cytosolic p53. Regulators of the MDM2–p53 interaction include the AKT/mTOR pathway, ARF, and c-MYC. Downstream transcriptional targets of p53 serve to arrest cell-cycle progression and induce cellular apoptosis. Multiple sites of action are targeted by MDM2 and MDM2–p53 inhibitors, which decrease p53 degradation in tumors with wild-type p53. Created with BioRender.com.

The tumor-suppressor protein p53 is a critical regulator of cellular processes including division, DNA repair, apoptosis, and senescence. Under physiologic conditions, p53 is maintained at low levels to facilitate appropriate cellular turnover (65). Activity of p53 is primarily regulated by MDM2, an E3 ubiquitin ligase that promotes p53 proteasomal degradation (**Figure 3**) (66). Loss-of-function mutations in MDM2 potentiate p53 acetylation and protein stability, resulting in upregulated downstream effectors (67). Conversely, *MDM2* amplification is associated with reduced p53 activity and has been implicated in multiple cancers including DDLPS (68, 69).

The MDM2 protein contains several structural elements that are necessary to inhibit p53. MDM2 physically interacts with p53 through its NH_2_ terminal domain, which forms a hydrophobic cleft that binds a helical region of the p53 transactivation domain (70). This conceals the transcriptionally active domain of p53 from co-regulatory proteins through a key-and-lock configuration. Additionally, MDM2 contains a C-terminus RING (zinc-finger) domain that ubiquitinates p53 at six separate lysine residues along its C-terminus, marking it for proteasomal degradation (71, 72). Nuclear localization signal (NLS) and nuclear export signal (NES) domains allow for the shuttling of MDM2 into the nucleus as well as into the cytoplasm for protein turnover, respectively (73). Because of this import/export function, MDM2 has been observed in both the nucleus and cytoplasm. In addition, the function of MDM2 is partly dependent upon the activity of its homolog, MDMX, which shares structural similarity within the N-terminus p53-binding domain (74). MDMX attenuates p53 activity through heterodimerization with MDM2 resulting in protein complex stabilization (75, 76).

Under physiologic conditions, DNA damage attenuates the MDM2–p53 interaction by two primary mechanisms. First, MDM2 can undergo inactivating kinase-dependent phosphorylation. The phosphorylation status of the central acidic and C-terminal RING domains of MDM2 are associated with the efficacy of MDM2-mediated p53 degradation (77). It has been shown that the ataxia telangiectasia-mutated (ATM) kinase, itself activated primarily by DNA double-stranded breaks, phosphorylates both the serine 395 residue within the central acidic domain and five sites near the C-terminal RING domain of MDM2, impairing MDM2-mediated nuclear exportation and the proteosomal degradation of p53 (78, 79). Similar inactivating kinase functions have been described for glycogen synthase kinase 3β, casein kinase 1, and casein kinase 2 (80–82). Second, MDM2 can undergo proteasomal degradation *via* self-ubiquitination. Oxidative by-products of DNA damage stimulate ubiquitin-activating and conjugating enzymes that drive MDM2 self-ubiquitination, which is dependent on the C-terminal RING finger domain (83, 84). DNA damage, particularly dsDNA breaks, also stimulate the activation of other E3 ligases that target MDM2 such as P300-CBP-associated factor (PCAF), a histone acetyltransferase and transcriptional co-activator of p53 (85, 86). Similarly, it has also been shown that phosphorylation of MDM2 by casein kinase 1 promotes SCF^β-TrCP^-dependent MDM2 ubiquitination and turnover (87).

Other posttranslational modifications that influence MDM2 regulation have been identified that may represent actionable targets for directed therapy. The addition of a small ubiquitin-like modifier (SUMO) protein to lysine residues, termed SUMOylation, occurs in response to DNA damage and abrogates the ability for MDM2 to bind p53, resulting in increased cellular p53 protein levels (88, 89). More recent work has characterized lysine modification by the small ubiquitin-like molecule, NEDD8. Watson et al. observed that NEDD8 stabilizes MDM2, termed NEDDylation, *via* binding to the MDM2 RING domain and preventing ubiquitination (90). The authors additionally identified that the NEDD8-specific isopeptide, NEDP1, which serves to de-NEDDylate MDM2, was induced by doxorubicin *in vitro*. NEDP1 activity was associated with an increased auto-ubiquitination of MDM2, a reduction in detectable MDM2 protein, and an increase in p53 protein expression by Western blot. As these findings indicate, there are multiple MDM2–p53 regulators, many of which require further investigation, and some of these regulators may potentially serve as useful targets for combination therapies.

## MDM2 cross talk with oncogenic pathways

The MDM2–p53 axis is also regulated by multiple, parallel oncogenic signaling pathways implicated in sarcomagenesis, and these pathways might represent valuable targets for combination therapeutic strategies in DDLPS. Among these, ARF-mediated MDM2 inhibition is well described. ARF, also referred to as p14ARF in humans, is encoded from the partially open reading frame *p14ARF* located on the *CDKN2A* locus and acts as a direct inhibitor of MDM2 (91, 92). Specifically, the N-terminal domain of p14ARF binds to MDM2, sequestering MDM2 to the nucleus (93, 94). Epigenetic alterations in *p14ARF* have been implicated in oncogenesis, particularly myxoid and pleomorphic sarcomas. Davidović et al. identified epigenetic silencing *via* methylation of the promoter region of *p14ARF* in 83% and 50% of myxoid and pleomorphic sarcoma samples, respectively (95). Similarly, Oda et al. identified hypermethylation of the *p14ARF* promoter with a subsequent reduced expression of the p14ARF protein and an overexpression of p53 in their cohort of round-cell liposarcoma samples (96). Variable levels of p14ARF expression have been observed in DDLPS (97). Taken together, p14ARF epigenetic silencing might represent a valuable target for affecting MDM2 function in sarcoma.

The proto-oncogene *MYC*—and its product c-MYC—is another critical regulator of MDM2 function *via* ARF signaling (98–100). c-MYC is a transcription factor that regulates numerous downstream targets supporting cellular survival and proliferation (101, 102). Under physiologic conditions, c-MYC activation has been observed to induce the expression of p14ARF, which in turn inhibits MDM2 (103, 104). However, the aberrant overexpression of c-MYC has been associated with a reduced expression of p53 by IHC and has been identified in numerous human cancers (105). Furthermore, high levels of c-MYC have been closely associated with hypermethylation of *CDKN2A* and loss of p14ARF, suggesting that overexpression of c-MYC may contribute to enhanced MDM2 activity (106, 107). Despite these data, there is a paucity of studies corroborating these findings in DDLPS. In this regard, Kim et al. used *in vitro* mesenchymal stem cells to co-express MDM2 and CDK4 in cell lines containing several known oncogenic mutational signatures, including stabilized c-MYC resistant to proteasomal degradation. The authors noted that c-MYC stabilized cell lines overexpressing MDM2 and CDK4 developed DDLPS like-morphology *in vitro (* 108). These findings were recapitulated *in vivo*, wherein nude mice that were inoculated with c-MYC-stabilized cell lines overexpressing MDM2 and CDK4 exhibited tumor growth with dedifferentiated lipoblasts on histologic analysis. This tumor growth was significantly larger and more accelerated when compared with nude mice harboring cell lines that lacked c-MYC stabilization. Taken together, these data suggest that c-MYC dysregulation in the context of MDM2 overexpression might influence lipoblast dedifferentiation and warrants further investigation in human DDLPS.

Finally, the aberrant activation of the phosphatidylinositol 3-kinase/AKT/mammalian target of rapamycin (PI3K/AKT/mTOR) pathway has gained increasing interest for its potential role in DDLPS. AKT is a serine/threonine kinase which, under physiologic conditions in response to both growth factor signaling and cell stress signaling, regulates a number of downstream cellular functions including cell-cycle progression, apoptosis, and DNA repair (109). AKT interacts with the MDM2–p53 pathway in two primary ways. First, in response to cellular growth factors, AKT phosphorylates MDM2 at one of two serine residues, causing nuclear localization and intranuclear binding of MDM2 to p53, and thereby facilitating p53 degradation (109, 110). Second, AKT has been observed to regulate the downstream transcription factor mTOR. The direct activation of mTOR by AKT *via* phosphorylation at the Ser2448 residue activates signaling pathways propagating cellular proliferation (111). AKT has additionally been observed to phosphorylate and subsequently repress tuberous sclerosis complex 2 (TSC2), which in turn suppresses mTOR signaling (112–114). Phosphorylation of mTOR results in an increased translation of MDM2, in turn promoting the degradation of p53 (115). Overexpression of this pathway is associated with unchecked progression through the cell cycle and has become increasingly identified as a driver of oncogenesis in DDLPS. Ishii et al. identified a significantly increased level of *mTOR* activation in DDLPS when compared with WDLPS by PCR, which was correlated with an increased mTOR expression by IHC (116). Using an *in vitro* model, the authors then cultured DDLPS cell lines with the mTOR inhibitor RAD001 and observed a decrease in cellular proliferation by 20%. Similar findings were observed by Gutierrez et al. wherein the authors documented evidence of aberrant AKT pathway activation *via* detection of phosphorylated Ser473-AKT in 47% of DDLPS samples (117). Tumor growth *in vitro* was abrogated by BEZ235, an inhibitor of the AKT pathway, suggesting that aberrant AKT activation influences DDLPS proliferation.

Taken together, these data illustrate the complex interplay of parallel oncogenic pathways that impact MDM2–p53 signaling in DDLPS. Co-inhibition of these pathways might be an effective strategy for addressing signaling redundancy and improving response rates to MDM2-targeted therapy.

## Targeting the MDM2 pathway: drug design and preclinical studies

Microscopic and genetic investigations of DDLPS over the last three decades have provided strong supporting evidence to target the MDM2 pathway (118, 119). *MDM2* amplification is associated with worse clinical outcomes, including a significantly shorter time to disease recurrence and reduced overall survival following oncologic resection (120). *MDM2* amplification is also associated with reduced sensitivity to doxorubicin chemotherapy (121). Accordingly, this oncogenic pathway has garnered significant interest as a therapeutic target.

Regarding drug design, MDM2–p53 binding is characterized by hydrophobic amino acid interactions for an energetically relaxed state (122). The p53 binding interface is located within the NH2 terminal transactivational domain, which contains an amphipathic alpha-helix with multiple hydrophobic residues (aa 18-26) (123). Using X-ray crystallography, Phe19, Trp23, and Leu26 within this helix have been identified as critical to stable binding of MDM2 (70). Conversely, the MDM2 binding interface is located within the NH2 terminal domain (aa 25–109), which creates a structural cleft that allows for the intercalation of hydrophobic residues on the p53 amphipathic helix (124). Within the crystal structure of MDM2, residues Gly58, Glu68, Val75, and Cys77 appear essential to p53 binding (125). The characterization of this binding interaction has led to the development of multiple synthetic compounds, most of which are defined by aromatic structures with amphipathic features that mimic the MDM2–p53 protein–protein interaction.

One group of such compounds includes Nutlins, which are *cis*-imidazoline analogs that rely on two bromophenyl groups and an ethyl ether side chain to mimic the p53 residues Trp23, Leu26, and Phe19, respectively. Nutlins have been extensively studied in preclinical models. Vassilev et al. utilized wild-type p53 and mutant p53 cell lines to demonstrate that specific Nutlin enantiomers, primarily Nutlin-3a, initiated apoptotic caspase activation in 45% of *MDM2*-amplified osteosarcoma cell lines at 48 h (125). Subsequent *in vivo* mouse xenograft models bearing the same wild-type p53 osteosarcoma cell lines were treated with Nutlin-3, which was associated with a 90% inhibition of tumor growth relative to vehicle controls. In a separate analysis, Tovar et al., using both *in vitro* cell lines and xenograft-bearing mice, demonstrated that RG7112, a second-generation Nutlin, induced a dose-dependent blockade of cell-cycle progression that was associated with increased p53 protein expression (126). Additional preclinical testing of Nutlin-3 has been examined in hematologic malignancies with *MDM2* amplification including multiple myeloma, acute myeloid leukemia, and chronic lymphocytic leukemia (127–129). It has also been reported that Nutlin-3a is able to induce p53-independent mechanisms of cell death by enhancing the stability of the tumor suppressor, p73, a member of the p53 family with pro-apoptotic activities (130). Additional p53-independent regulatory targets of MDM2 have been reported such as pRb and E2F/DP (131).

SAR405838, a non-Nutlin small molecular inhibitor of MDM2, has also been investigated in preclinical experiments and clinical trials. Wang et al. administered SAR405838 to mice bearing xenografted leukemia and solid tumor implants (132). The authors identified intracellular accumulation of p53 within tumor tissue and increased transcriptional activity of wild-type p53, which was associated with an increase in pro-apoptotic proteins and cell-cycle arrest. Bill et al. recapitulated these findings *in vitro*, showing that MDM2 inhibition with SAR405838 induced cell-cycle arrest and apoptosis in wild-type p53 human DDLPS tumor cells, which was associated with enrichment of p53-mediated gene expression patterns (133). The authors also showed that SAR405838 was associated with significant reductions in tumor volume in DDLPS xenograft-bearing mice.

HDM201, a more recent MDM2 inhibitor, has shown improved potency and selectivity. Preclinical data from Jeay et al. demonstrated cell growth inhibition in p53 wild-type, *Mdm2*-amplified osteosarcoma cells utilizing both continuous and pulsed HDM201 treatment methods (15). Compared with controls, HDM201 treatment *in vitro* was associated with induction of p53 downstream markers. This included a 16-fold induction of *P21* mRNA, which encodes for a protein that is a potent cyclin-dependent kinase inhibitor that plays a crucial role in DNA-damage-related cell-cycle arrest (134, 135). *Mdm2*-amplified osteosarcoma cells were then xenografted into rats, and the animals were treated with HDM201. The investigators observed induction of multiple cell-cycle arrest-related genes in a dose-dependent manner. These included *Puma*, *p21*, and *Gdf*, which are well-described downstream proapoptotic targets of p53 that contribute to cell-cycle arrest and apoptosis (136, 137). High-dose HDM201 was associated with tumor cellular apoptosis and complete tumor regression, which was sustained in all rats for 30 days after stopping treatment. The authors performed an additional study using *Mdm2*-amplified WDLPS cells that were xenografted into mice, and they observed similar rates of tumor regression in response to HDM201 treatment.

Finally, the efficacy of HDM201 in preclinical studies may be partly due to its effect on the tumor immune microenvironment. Wang et al. investigated HDM201-mediated immunologic changes within the tumor microenvironment using an immunocompetent, syngeneic mouse tumor model (16). HDM201 treatment was associated with increased tumoral levels of CD103+ antigen-presenting dendritic cells, which are thought to play a critical role in tumor antigen presentation and priming of cytotoxic T lymphocytes (138). The authors additionally observed an increase in Tbet+Eomes+CD8+ cytotoxic T cells, a subset of cytotoxic T cells which have demonstrated potent cytotoxic activity with an exhausted phenotype (139, 140). T-cell exhaustion has been closely associated with response to anti-PD-1 therapy, particularly in combination with chemotherapy or other immunotherapeutics (141, 142). The authors therefore combined HDM201 with an anti-PDL-1 antibody, which was associated with significantly longer survival when compared with HDM201 monotherapy. Antitumor activity with combination therapy was abrogated with *Tp53* knockout, indicating that HDM201 treatment was required for induction of an immunologic response. These data suggest that modulation of the tumor microenvironment may synergize with MDM2-targeted therapies; as such, this remains an area of ongoing investigation.

## Clinical trial results for MDM2 pathway inhibitors in liposarcoma and solid tumors

Despite the promising preclinical data, MDM2 inhibition has yielded mixed results in clinical trials (**Table 1**). Ray-Coquard et al. reported findings from a phase I clinical trial evaluating RG7112, in which patients with chemotherapy-naïve primary or relapsed WDLPS and DDLPS received neoadjuvant treatment prior to surgical resection (14). Twenty patients were enrolled, of which 18 were p53 wild-type and 14 had *MDM2* amplification identified *via* silver *in-situ* hybridization. Nineteen of 20 patients completed at least one preoperative treatment cycle. Four patients received only one cycle of treatment; two were discontinued for neutropenia and thrombocytopenia, one for progressive disease and one for phlebitis. Five patients received two cycles, and 10 patients received three cycles. All patients had at least one adverse event, and there were 12 serious adverse events in eight patients. The authors raised concerns about gastrointestinal and hematologic toxicity with long-term usage. Measured p53 protein concentrations were significantly increased 4.86-fold from pretreatment levels, and p21 protein concentrations were significantly increased 3.48-fold. However, treatment response as measured by Response Evaluation Criteria in Solid Tumors (RECIST) criteria included only one confirmed partial response, stable disease in 14 patients, and progressive disease in five patients (all DDLPS).

**Table 1:** List of clinical trials investigating MDM2 inhibition in liposarcomas or other solid tumors.

Compound	Drug combination	Tumor type	Trial phase	Status	Trial identifier	Trial results
***ALRN-6924***	–	Solid tumors	I	Recruiting	NCT03654716 (143)	Pending
	Paclitaxel	Solid tumors	I	Recruiting	NCT03725436 (144)	Pending
	–	Solid tumors, lymphomas	I/II	Completed	NCT02264613 (145)	Overall disease control rate of 59% (146)
***AMG 232***	Radiation	Soft-tissue sarcoma	I	Recruiting	NCT03217266 (147)	Pending
	–	Solid tumors, multiple myeloma	I	Completed	NCT01723020 (148)	Best response of stable disease in 66% of patients (45/68); stable disease in 7/10 DDLPS (149)
***APG-115***	Toripalimab	Liposarcomas, solid tumors	I/II	Recruiting	NCT04785196 (150)	Pending
	Pembrolizumab	Solid tumors, metastatic melanoma	I/II	Recruiting	NCT03611868 (151)	Antitumor effects in multiple tumor types; no response in liposarcoma (n=14) (152)
	–	Solid tumors, lymphomas	I	Completed	NCT02935907 (153)	Best response of stable disease in 21.4% of patients (154)
***ASTX295***	–	Solid tumors	I/II	Recruiting	NCT03975387 (155)	Pending
***BI-907828***	Doxorubicin	Dedifferentiated liposarcomas	–	Recruiting	NCT05218499 (156)	Pending
	–	Solid tumors	I	Recruiting	NCT03449381 (157)	Disease-control rate of 87.5% (28/32) DDLPS patients (158)
	BI-754091, BI-754111	Solid tumors	I	Recruiting	NCT03964233 (159)	Pending
***CGM097***	-	Solid tumors	I	Completed	NCT01760525 (160)	Overall disease control rate of 39% (161)
***DS-3032b***	Trabectedin	Dedifferentiated liposarcomas	III	Recruiting	NCT04979442 (162)	Pending
	-	Solid tumors	II	Recruiting	NCT05012397 (163)	Pending
	-	Solid tumors, lymphomas	I	Completed	NCT01877382 (164)	Best response of stable disease in 60% of patients (165)
***HDM201***	Ribociclib	Liposarcomas	I/II	Completed	NCT02343172 (166)	Partial response in 4% of patients (3/74), stable disease in 51% (38/74) (167)
	Pazopanib	Soft-tissue sarcomas	–	Not yet recruiting	NCT05180695 (168)	–
	–	Solid tumors	I	Completed	NCT02143635 (169)	Overall response rate of 10.3% (170)
	Ribociclib	Solid tumors	II	Recruiting	NCT04116541 (171)	Pending
***RG7112***	-	Liposarcomas	I	Completed	NCT01143740 (172)	20 patients enrolled; results pending
	Doxorubicin	Soft-tissue sarcomas	I	Completed	NCT01605526 (173)	Best response of stable disease in 50% of patients (174)
	-	Solid tumors	I	Completed	NCT00559533 (175)	106 patients enrolled; results pending
	-	Solid tumors, lymphomas	I	Completed	NCT01164033 (176)	Best response of stable disease in 8% of patients (177)
***RO5503781***	–	Solid tumors	I	Completed	NCT03362723 (178)	48 patients enrolled; results pending
	–	Solid tumors	I	Completed	NCT02828930 (179)	Best response of stable disease in 12.5% of patients (180)
	–	Solid tumors	I	Completed	NCT01462175 (181)	Best response of stable disease in 30.6% of patients (182)

DDLPS, dedifferentiated liposarcoma.

Similar findings were observed in a phase I trial for SAR405838, in which patients with locally advanced or metastatic, medically refractory solid tumors with a p53 wild-type or a marginal p53 mutation (<40%) received SAR405838 monotherapy (13). Of the 74 patients enrolled, the most common primary tumor type was liposarcoma (n = 35, 47%). The study contained a second expansion cohort composed of 21 patients with DDLPS to determine the maximum tolerated dose (MTD). Of this expansion cohort, 89% of tumors exhibited *MDM2* amplification and no *TP53* mutations were observed. Regarding the total cohort, the most frequent treatment-related adverse effects were nausea (59%), fatigue (58%), and vomiting (42%). Serious adverse effects occurred in 30% of patients, including thrombocytopenia (8%), neutropenia (4%), and lymphopenia (16%). Eleven patients required drug discontinuation, and 12 required drug dose modification. In this study, there were no objective responses, although 58% of subjects had a stable disease per RECIST criteria. On subgroup analysis of the primary cohort, DDLPS patients had a higher percentage of stable disease (22/31, 71%). The MTD expansion cohort of DDLPS patients similarly had a 56% rate of stable disease, and progression-free survival at 3 months was 32%. The authors concluded that despite the lack of objective response, evidence of disease stabilization and p53 activation in the majority of patients warranted further evaluation, particularly for combination regimens.

HDM201 was recently investigated in a phase I, multicenter, open-label trial (170). In total, 115 patients with treatment-refractory locally advanced or metastatic solid tumors and 93 patients with relapsed or refractory hematologic malignancies were included. Treatment-related adverse events, most commonly gastrointestinal disorders and cytopenias, were observed in 90% patients with solid tumors and 88% of patients with hematologic malignancies. Approximately 40% of patients experienced at least one adverse event that required dose adjustment or interruption. Baseline biomarker status was evaluated in 48 patients using the FoundationOne panel, which demonstrated *MDM2* amplification in 16 (33%). *MDM2* amplification was more prevalent in patients who experienced either a partial response or stable disease (53%) compared with patients with progressive disease (23%). Among patients with progressive disease, mutations in *CDKN2A*, *ATRX*, *KRAS*, *AKT1*, and *CDK6* were also observed. For solid tumors, the objective response rate was 3.5% and the stable disease rate was 36.5% by RECIST criteria. Twelve patients with liposarcoma were treated in a dose-expansion phase of the study. Of these, one patient experienced a partial response (8%) and nine (75%) patients had a stable disease. The median progression-free survival for these 12 patients was 5.6 months, and one-third of patients had a stable disease for greater than 6 months. Taken together, despite the limited efficacy of HDM201 among the total cohort of patients with solid tumors, the authors noted that patients with liposarcoma in the expansion group experienced noticeable disease control. Favorable responses among those treated for hematologic malignancies were also observed. In conclusion, the authors recommended further investigation of HDM201 therapy, alone or in combination; there are currently two ongoing HDM201 clinical trials for patients with liposarcoma.

Finally, phase I trials investigating the MDM2 inhibitors CGM097 and DS3032b have been studied in solid tumors and lymphoma. CGM097 was evaluated in 51 patients with locally advanced or metastatic, *TP53* wild-type solid tumors (161). While no dose-limiting toxicities were reported, delayed-onset thrombocytopenia, nausea, leukopenia, vomiting, and fatigue were all recorded at incidences of greater than 20%. Stable disease was observed in 19/51 (37%), and one melanoma patient experienced a partial response as measured by RECIST criteria. The trial investigators observed evidence of p53 reactivation *via* induction of downstream molecular targets but without an associated clinically significant tumor response. DS3032b was evaluated in a phase I study of patients with WDLPS/DDLPS, solid tumors, and lymphomas (165). In this study, 63% of patients had been treated with three or more prior therapies and 87% of tumors were documented *TP53* wild types. Of the 94 patients enrolled in the study, 60% of patients achieved stable disease for a median duration of 6.7 months with three partial responses observed among patients with DDLPS, synovial sarcoma, and squamous cell lung cancer by RECIST criteria. Adverse events included thrombocytopenia (61%) and neutropenia (28%), with 8% of patients experiencing dose-limiting toxicities.

Inhibition of co-activators of the MDM2–p53 pathway has also been investigated in clinical trials. Of these, mTOR inhibition is well studied, and initial trials examining mTOR inhibition monotherapy using either temsirolimus or ridaforolimus yielded limited clinical efficacy. Okuno et al. conducted a phase II single-institution clinical trial examining the utilization of temsirolimus monotherapy in patients with advanced STS. Of the 40 patients that were enrolled, 95% experienced progression while on treatment and median overall survival was 7.6 months (183). Analysis of posttreatment peripheral blood samples demonstrated suppressed mTOR signaling in approximately 66% of patients, indicating adequate therapeutic levels of temsirolimus. Similar findings were observed in subsequent clinical trials. Chawla et al. conducted a phase II multicenter clinical trial examining the use of ridaforolimus monotherapy in patients with advanced STS. The median overall survival in their cohort was 10 months, and the overall response rate was 1.9% as determined by RECIST 1.1 criteria (184). Additionally, 98% of patients reported some form of adverse event, with 11% of patients discontinuing therapy due to poor tolerance of side effects.

Due to the disappointing results of mTOR inhibitor monotherapy and guided by preclinical observations that tumor cell growth of breast and prostate cancer cell lines *in vitro* was abrogated when treated with combination mTOR/MAP-kinase inhibition, several early-phase clinical trials examined combination therapy with mTOR inhibition (107, 117, 185, 186). Eroglu et al. examined MAP-kinase inhibition monotherapy versus combination mTOR/MAP-kinase inhibition among a cohort of STS patients in a randomized, phase II clinical trial (185–187). There was no significant difference in progression-free survival between the two treatment arms; however, the authors noted that combination therapy was associated with significantly improved median PFS in the leiomyosarcoma subgroup. Based on these data, there was increased interest in the combination of mTOR inhibitors as an adjunct to first-line chemotherapy for the treatment of STS. Trucco et al. conducted a phase II clinical trial examining mTOR inhibition with temsirolimus in combination with doxorubicin in patients with advanced, non-resectable STS. The median PFS for the study population was 10.3 months, and the best response was partial response in 13% of patients as determined by RECIST 1.1 criteria (188). The authors did not report histology-specific outcomes but did note that median PFS was slightly better with combination therapy when compared with mTOR inhibitor monotherapy. Taken together, these data suggest that mTOR inhibition may provide some clinical benefit in select histologic subtypes when combined with other systemic therapies. However, the utility of mTOR inhibition in DDLPS remains unclear and the combination of mTOR and MDM2 inhibition has not been evaluated.

c-MYC represents another co-activator of MDM2, and direct c-MYC inhibitors have demonstrated low cellular affinity and minimal *in vivo* potency (189–191). This is thought to be a result of difficulties with targeting the transactivation domain. Nuclear magnetic resonance studies have been used to demonstrate that the transactivation domain of c-MYC exhibits a heterogenous conformational space depending on the phosphorylation status of its N-terminus (192). However, transient structural elements of the MYC protein stabilize when bound to other cofactors, notably Aurora kinase A (AURKA) (193). Heterodimeric modeling has allowed for the development of indirect MYC inhibitors through targeted AURKA inhibition (194). In this regard, Dickson et al. conducted a multicenter phase II clinical trial investigating the use of AURKA inhibitor MLN8237 in patients with advanced or metastatic STS. The overall response rate was 2.8%, and the median PFS was 11.7 weeks (195). Among liposarcoma patients specifically, median PFS was 13 weeks and median OS was 68 weeks. The authors identified that 73% of liposarcoma patients were progression-free at 12 weeks, which they suggest is promising based on previous reports designating favorable drug response in STS as 40% progression-free at 12 weeks (196). Of note, p53 activity was not significantly changed in the posttreatment correlative specimen analysis of DDLPS as determined by Western blot. Given these findings, there is ongoing work to elucidate the clinical benefit of various c-MYC cofactor inhibitors.

There have also been studies investigating cyclin-dependent kinase (CDK) inhibitors as modulators of the p53 pathway. Specifically, CDK4/6, which share overlapping function and structural homology, have been robustly examined as a targetable modulators of the cell cycle (197). CDK4/6 phosphorylates the retinoblastoma tumor-suppressor protein (Rb1), which in turn dissociates Rb1 from the transcription factor E2F and allows progression of the cell cycle from the G1 to S phases (198). CDK4/6 has been identified as a downstream target of the AKT/mTOR pathway, suggesting that CDK4/6 may have synergistic effects with signaling molecules co-expressed in the AKT-MDM2 axis (199). Additionally, *CDK4/6* is often upregulated alongside *MDM2* due to 12q13-15 amplification, although not as frequently; Binh et al. demonstrated *CDK4* amplification in 90% of DDLPS samples when compared with the near 100% prevalence of *MDM2* amplification (200). Taken together, CDK4/6 inhibition represents an alternative mechanism for p53 reactivation in addition to MDM2 inhibition.

Clinical trials investigating the use of CDK4/6 inhibitor palbociclib has yielded modest clinical benefit. Dickson et al. conducted a phase II, non-randomized clinical trial in which patients with advanced WDLPS and DDLPS received palbociclib. Of the 28 patients, 57% achieved progression-free survival at 12 weeks and one patient achieved durable complete response 2 years after treatment (201). However, the authors did not include histology-specific outcomes and close to 80% of patients experienced tumor growth while on treatment. Due to the disappointing results of CDK4/6 inhibitor monotherapy, there has been increasing interest in combination CDK4/6 and MDM2 inhibition. Razak et al. conducted a phase Ib proof-of-concept trial in which patients with advanced WDLPS and DDLPS received concomitant siremadlin, a selective MDM2 inhibitor, with ribociclib, a selective CDK4/6 inhibitor. In this study, no patients achieved complete response and three patients achieved partial response (202). The overall disease control rate remained poor at 27% among the cohort, and one patient died of treatment related hematotoxicity.

In summary, the majority of targeted therapy studies to date in DDLPS demonstrate inferior clinical performance when compared with the estimated objective response rate of 26% for first-line anthracycline-based chemotherapy for DDLPS (203). Therefore, the majority of studied targeted inhibitors are currently not recommended as an adjunct to systemic therapy. Per NCCN guidelines, palbociclib may have utility under certain circumstances (204). Despite these discouraging results, it should be noted that most of these studies were performed in advanced-stage disease refractory to multiple lines of therapy, and the therapeutic potential of combination therapy requires further investigation.

## Mechanisms of resistance to MDM2 inhibition

Given the poor performance of MDM2 inhibitors in clinical trials for solid tumors, certain research efforts have shifted focus toward elucidating mechanisms of resistance. One commonly identified mechanism of resistance involves mutation in the *TP53* gene that either renders the protein inactive or mitigates binding affinity for MDM2. Jung et al. evaluated liposarcoma tumor biopsy samples from patients treated in the SAR405838 trial over multiple time points, including a pretreatment baseline and every 6 weeks thereafter for 36 weeks (205). Upon examination of circulating cell-free DNA, the authors identified 26 acquired *TP53* missense mutations during the study interval. Variants appeared within 6 weeks after treatment initiation and occurred with increasing frequency with cumulative therapy. Most mutations were in the p53-DNA-binding domain, rendering the tumor suppressor ineffective. The authors concluded that MDM2 inhibition exerted selective pressure on malignant cells, driving clonal expansion of lineages with inactivating *TP53* mutations. This observation corresponded clinically with an early, modest clinical benefit that diminished over time.

In a separate study, Chapeau et al. performed a genetic screen to identify additional mechanisms of resistance to MDM2 inhibition (206). Using a piggyBac insertional mutagenesis system, the authors performed mutagenesis on genetically modified *Arf -/-* mice (207). *CDKN2A* deletion with subsequent p14ARF inactivation results in MDM2 activation in certain human malignancies. Tumors derived from these mice were allografted, and mice were then treated with the MDM2 inhibitor, HDM201. In total, 87 genes were significantly altered by mutagenesis. Sixteen of 21 tumors initially responded, but all relapsed over time. Refractory tumors displayed outgrowth of unique subclones resistant to MDM2 inhibition. Among these, multiple genetic mechanisms of acquired treatment resistance were identified, including both somatic and insertional changes. Loss-of-function mutation in *Tp53* was observed in 54% of resistant tumors, and upregulation of antiapoptotic B-cell lymphoma-extra-large (*Bcl-xL*) and *Mdmx* gain-of-function mutations were also frequently identified. On subsequent combination therapy testing, the authors observed an additive effect using a *Bcl-xL*- selective inhibitor (ABT-263) in combination with MDM2 inhibition in 35/138 *Tp53* wild-type cell lines, providing further support for investigation of combination therapies.

*MDMX* upregulation and gain of function represent another mechanism of resistance to MDM2 inhibition. Although MDMX does not have E3 ubiquitin ligase activity, it shares structural homology with MDM2 in the NH2 terminus p53-binding cleft, and it is able to independently sequester the p53 transactivational domain (208–210). MDMX also contains a C-terminus RING finger domain that allows for binding and stabilization of the MDM2 protein, and both functions contribute toward p53 inactivation (211). These effects have been demonstrated *in vitro*, in which human fibroblast cells were transformed to overexpress MDMX, resulting in acquired resistance to Nutlin-3a-mediated MDM2 inhibition. Conversely, Nutlin-3a treatment was rescued by siRNA knockdown of *MDMX*. Deletion of the *MDMX* C-terminus RING finger domain similarly rescued Nutlin-3a treatment, suggesting its structural role in MDM2 binding and stabilization. Based on these findings, combination approaches to simultaneously address MDM2 and MDMX function may improve treatment response rates and mitigate acquired resistance.

Stapled peptides, which are composed of stabilized alpha-helical peptides, are one potential strategy to target shared protein–protein interactions between MDM2–p53 and MDMX–p53, acting as a dual MDM2/MDMX inhibitor (212). ALRN-6924 was the first clinical drug of this type to be studied in a phase I, open-label, multicenter clinical trial (213). Seventy-one patients were enrolled, of which 17 had sarcomas, 60 had other solid tumors, and four had lymphoma. Ninety percent of tumors were *p53* wild types. In total, 41 patients were evaluable for treatment efficacy, and of these, 24 patients (59%) demonstrated disease control by RECIST criteria. Four patients achieved a response: two complete responses and two partial responses; one of the partial responses was liposarcoma of unspecified subtype. The median duration of clinical benefit was 7.5 months. There were 10 patients with *p53* wild-type and *MDM2* tumor amplification, of which five patients (71%) achieved disease control. A trend was observed in which patients with lower expression levels of MDMX were more likely to achieve disease control than were patients with higher MDMX levels, although this association did not reach statistical significance. ALRN-6924 showed a good safety profile with the most common toxicities consisting of mild to moderate nausea, fatigue, vomiting, and myelosuppression. The limited side effect profile combined with improved rates of disease control provides rationale for further study (213).

Genomic and transcriptomic analyses of MDM2-resistant cells have revealed additional potential resistance mechanisms (214–216). *N-RAS*, *MAPK/ERK*, *IGFBP1*, and *NF-kB*, which are known drivers of cell proliferation and survival, are frequently upregulated in response to MDM2 inhibition. Increased drug export is an additional form of treatment resistance observed with MDM2 inhibitors. Grigoreva et al. compared the expression levels of the *ABC* gene encoding P-glycoprotein export protein in colon cancer cell lines treated with Nutlin-3a compared with controls and demonstrated that resistant lines exhibited 3–19-fold increased mRNA expression, which correlated with increased protein expression by Western blot (217). The authors utilized a fluorescent dye that binds to the intracellular H-site of P-glycoprotein and is actively released from cells. In so doing, the authors observed a two-fold increase in fluorescence intensity within wild-type cells when compared with resistant cell lines, suggesting greater intracellular accumulation of dye within non-resistant cell lines and increased dye efflux within chemoresistant cell lines. Taken together, these data suggest that increased P-glycoprotein-mediated efflux may contribute to drug resistance. While the molecular pathogenesis of these mechanisms requires further characterization and validation, these findings indicate that there are myriad ways in which cancers can evade targeted therapy. In this context, there has been increasing interest in combination therapy to overcome common mechanisms of resistance.

To date, there has been some success with combination techniques, mostly in preclinical studies. Saiki et al. conducted a cell-based screen to identify compounds that synergize with MDM2 inhibition in *TP53* wild-type cells (218). In total, 13/1,169 library compounds were potential candidates, including PI3K/MAPK inhibitors, BH3 mimetics, BCR-ABL kinase antagonists, and HDAC inhibitors. Triple-combination studies using MDM2, PI3K, and BRAF/MEK inhibitors demonstrated even greater suppression of cell growth than all permutations of two-way combinations. In a separate *in vitro* analysis, it was shown that combination MDM2 inhibition plus doxorubicin was associated with significantly reduced tumor growth compared with doxorubicin monotherapy, suggesting that the addition of MDM2 inhibition to conventional cytotoxic therapy might also be beneficial (120). Finally, 12q13-15 co-amplification of *CDK4* and *MDM2* may represent therapeutic targets for combination therapy. While mixed results have been observed in preclinical application of CDK4/MDM2 combined targeting, there is an ongoing multicenter phase II clinical trial investigating co-administration of HDM201 with LEE011, a known CDK4 inhibitor (NCT02343172) (219, 220). In all instances of proposed combination strategies, the main barrier to implementation remains a concern for significant clinical side effects, which can be observed with even single-agent use of targeted treatments.

## Key implications and future directions

When thinking to the future of targeted therapy for DDLPS, the findings from this review imply several key considerations. First, while MDM2 amplification is convincingly implicated in DDLPS oncogenesis, further investigation is necessary to better assess its importance in established tumors. In clinically detectable disease, it is possible that MDM2 hyperfunction is non-essential given the relative ease for tumors to develop resistance mechanisms in response to targeted therapy. Further studies in this regard would help to better appraise the utility of continuing to pursue the MDM2 pathway as a pharmacologic target. Second, regarding mechanisms of resistance, based on the seminal studies by Jung and Chapeu et al., selection for p53 inactivating mutations is a common phenomenon to bypass targeted MDM2 inhibition in sarcoma. As a key regulator of cell-cycle arrest, inactivating p53 mutations further limit the potential for activation of tumor-suppressive programs. Thus, co-targeting of parallel, interacting oncogenic pathways including PI3K/AKT/mTOR, MYC, and Bcl-x would seem a reasonable approach to mitigating MDM2 bypass mechanisms. Third, regarding the use of targeted therapies, whereas there have been preliminary studies investigating combination approaches for sarcomas, these have often been based on drug availability in the setting of advanced disease and not necessarily designed for synergistic or cooperative function, likely limiting the potential for this approach. Furthermore, whereas increasing the combination of inhibited targets would potentially lead to greater efficacy, toxicity remains an ongoing and major challenge for targeted therapies. Most combinatorial studies to date involve two agents, but it is conceivable going forward to have studies with multiple drugs designed in a rational cocktail, toxicities permitting. In this regard, development of more accurate real-time biomarkers to measure treatment response in DDLPS—such examples might include cell-free DNA (cfDNA) and advanced radiomics—is essential helping to monitor for resistant clonal outgrowth with targeted agents. Finally, in the current era, targeted therapy inevitably fails for most malignancies, but the phenotypic impacts of targeted therapy remain poorly understood and could potentially be used to leverage other treatment modalities. For example, regarding MDM2 inhibition in DDLPS, by virtue of selecting for specific clonal populations with phenotypic features such as inactivating p53 mutations, important questions arise regarding the impact on tumor heterogeneity, sensitivity to cytotoxic therapy, or changes in immunogenicity. While still entirely hypothetical, characterizing patterns of iatrogenic tumor evolution are minimally described to date in DDLPS, and this would be potentially useful when thinking about the implications of MDM2 inhibition.

## Discussion

*MDM2* amplification in DDLPS represents an excellent therapeutic target in theory, although clinical applications have thus far been disappointing. For reasons that are not fully understood, MDM2 inhibition has been more successful in other malignancies such as acute myelogenous leukemia, chronic lymphocytic leukemia, and multiple myeloma, in which early-phase clinical trials have demonstrated efficacy (127–129). In both preclinical experiments and clinical trials for solid tumors, MDM2 inhibition has been associated with an increase in p53 protein expression, yet this observation has not been associated with improved outcomes such as tumor growth inhibition or longer survival. Findings from recent studies indicate that this observed discrepancy is a result of multiple factors, including clonal expansion of cell lines with inactivating *p53* mutations, augmentation of co-activators such as MDMX, and other unforeseen mechanisms of resistance (205). The tumor suppressor p53 is one of the key regulators of cell-cycle progression and is thus influenced by numerous signaling pathways beyond MDM2 (221, 222). As might be expected, MDM2 inhibition alone appears insufficient in the face of oncogenic redundancy.

Despite these challenges, MDM2-targeted therapies remain of interest in the treatment of DDLPS. Attempts to optimize the pharmacokinetics and dynamics of novel MDM2 inhibitors are underway. Novel drugs designed to simultaneously inhibit both MDM2 and MDMX represent an area of active research. In addition, alternative targets amplified on the 12q arm, including *CDK4*, *HMGA2*, and *YEATS4*, are also being investigated for combination therapies (223, 224). Advancements in peptide synthesis and cell-free DNA represent promising future directions in the targeted treatment and surveillance of these tumors. Taken together, these promising avenues of research will hopefully lead to less toxic and more efficacious treatments that mitigate the severity of this deadly disease.

## Author contributions

RT, BC CR, EK, EN, and DE performed data acquisition, data interpretation, and manuscript construction. All authors contributed to the article and approved the submitted version.

## Funding

Supported in part by grant from the National Institute of Health (T32CA009599 to RT). There are no other financial relationships related to the design or execution of this study.

## Conflict of interest

The authors declare that the research was conducted in the absence of any commercial or financial relationships that could be construed as a potential conflict of interest.

## Publisher’s note

All claims expressed in this article are solely those of the authors and do not necessarily represent those of their affiliated organizations, or those of the publisher, the editors and the reviewers. Any product that may be evaluated in this article, or claim that may be made by its manufacturer, is not guaranteed or endorsed by the publisher.
